# Meetings of Societies

**Published:** 1904-12

**Authors:** 


### MEETINGS OF SOCIETIES.

### Bristol Medico-Chirurgical Society.

*Annual Meeting, October 12th, 1904.*

After a vote of thanks to the retiring President (Mr. Paul Bush) had been unanimously passed, Dr. Reginald Eager took the chair, and gave an introductory address (see p. 289). Subsequently a cordial vote of thanks was given to Dr. Eager for his able address.

The Honorary Secretary, Mr. H. F. Mole, read his Annual Report. The balance in hand was £158 4s. 8d. on the ordinary account and £10 0s. 1d. on the *Journal* account.

The Society had to regret the loss of two members through death, viz. Drs. E. Crossman and T. Hill; the former was one of the oldest members of the Society. The report was adopted, and thanks given to the Secretary for his services.

The Editorial Secretary, Mr. James Taylor, read his Annual Report, which was followed by that of Mr. C. K. Rudge, the Honorary Librarian of the Medical Library. The latter report showed that the Bristol Medical Library now contained 20,764 volumes, and was in receipt of 227 current periodicals. These reports were adopted.

The following officers were chosen for the ensuing year:—President-elect, Mr. Dacre; Members of Committee, Dr. J.

Michell Clarke, Mr. Dacre, Dr. B. M. H. Rogers, Mr. James Taylor, Dr. Waldo, Dr. Watson Williams, and Mr. C. K. Rudge; and Members of the Library Committee, Mr. Rudge, Mr. Munro Smith, and Mr. T. Carwardine.

Dr. Shingleton Smith and Mr. Paul Bush were elected to represent the Society at the next International Medical Congress to be held in Lisbon in 1906.

---

*November 9th, 1904.*

Mr. Munro Smith in the chair.

Dr. Ogilvy showed a patient with **peculiar changes in the iris.** The patient, a man 22 years of age, had been struck in the left eye with a chip from a hammer when driving in a nail. The accident had taken place three years previously. The patient stated that for a fortnight after the accident vision was obscured; then it cleared up, and he went on with his work till six months ago without much trouble. Then he noticed his sight was failing, but had not sought advice till a month ago. The state of affairs when seen showed a pupil tied down all round, and with considerable opacity in the centre, but no active iritis. Under atropin the synechiæ broke down fairly easily, and it was then discovered that he had several nodules on the anterior capsule of the lens, bright orange in colour. These were at first taken to be spots of lymph left by the synechiæ; their colour, however, was brighter than those usually found in this condition. Their appearance resembled a condition sometimes found in cases where chips of steel or iron have remained embedded in the lens for a long time without causing any change, but which have sooner or later become oxidised, set up a definite opacity in this body, and eventually, if the lens has not been removed, have formed rusty fragments, leading to iridocyclitis and loss of the eye, with great danger to its fellow from sympathetic ophthalmia. In this eye, however, there was no cicatrix visible, nor any indication of the penetration of a fragment, and one was driven to the conclusion that these six or seven spots were merely tags of lymph, and their light orange-red colour was an abnormality.—Dr. James Taylor stated that if the spots were really "iron rust" the skiagraphic plate would undoubtedly show them up.—Mr. H. F. Mole asked if, owing to the extremely regular arrangement of the pigment, it was not more likely to be a deposit from the iris rather than rust from a foreign body.

Mr. A. W. Prichard read notes on the following cases:—First, a **case of mesenteric cyst.** The patient and specimen were shown. (For the full account of this case see page 332.) Secondly, a **case of intestinal obstruction from gallstone.** The large gallstone which had produced the obstruction was exhibited. It had been removed from the lower part of the jejunum; but the patient, aged 65, did not recover. The autopsy showed that the gall-bladder was adherent to the duodenum by adhesions, which were easily broken down, and that there was a large aperture of communication between the two viscera, through which the stone had passed. Both kidneys showed pyelonephritis. Thirdly, **a peculiar ovarian tumour** which had been removed by operation. It consisted of a large cyst connected with a solid mass, from which projected some papillomatous growths, and a large number of cysts of different sizes, and in the fresh specimen, of various colours. The growth was adherent to the bladder, and the pedicle was the left broad ligament. The patient did well.—Mr. ROGER WILLIAMS, discussing the first case, considered the specimen to be a remarkably good example of that rare form of omental cyst which, on account of its structural resemblance to intestine, had been well named "**enteroid.**" Such cysts were undoubtedly due to sequestration of "rests" of the evolving intestine, separated during the course of development. In this connection it was of interest to recall the fact that cysts of this kind were specially apt to arise from the part of the gut in the vicinity of the bile-papilla, and also from that in the vicinity of the vitelline duct: both of these localities were the sites of great developmental complication. In this case the presence of duodenal-like glandular structures and even Brunner's glands in the wall of the cyst, clearly pointed to its locality of origin—the vicinity of the bile-papilla. This was a region of great developmental complication; here it was that the fore and mid gut joined, and not as was usually stated at the pylorus. There was no special developmental complication at the pylorus. Developmentally regarded, the first part of the duodenum to within a short distance of the bile-papilla, really formed part of the stomach. It was in the vicinity of the bile-papilla that atresia and contractions were so often met with, and it was with malformations of this kind that the origin of these "enteroid" cysts were associated. The many ducts arising as outgrowths from this part of the bowel—abnormally evolving—probably had much to do with the causation of these conditions. He inquired whether these cysts communicated with the bowel by a pedicle or otherwise, and as to whether there were any signs of duodenal narrowing or deformity. In cases of this kind the cyst contents were generally of the nature of succus entericus. Referring to the **ovarian cystoma,** the speaker said that it was evidently a specimen of a rare and unusual kind of ovarian tumour of which it would be interesting to have a thorough examination made as to its precise structure. The grape-like, racemose appearance of the surface cysts was very unusual, and seemed to indicate that we had before us a specimen of "Rokitansky's tumour," a disease which was usually bilateral.—Mr. C. A. MORTON alluded to three cases of mesenteric or omental cysts he had met with and showed the specimens. In one a mesenteric cyst had been found in an inguinal hernia, and had made the hernia irreducible. A second he had considered to be an ovarian cyst before operation. Its contents had resembled liquid clay-coloured fæces. The third was situated in the transverse meso-colon in the right side, and had produced jaundice by pressure on the common bile duct.—Mr. Paul Bush commented upon the very rapid heart's action, still persisting, of the patient from whom Mr. Prichard had removed the mesenteric cyst. The boy was in other respects well, and there was no other obvious cardiac defect.—Mr. Munro Smith said that the microscopic structure of the cyst was that of the small intestine; two distinct muscular coats with a muscularis mucosæ and numerous large glands lined with columnar and pyramidal epithelium, like the glands in the pyloric end of the stomach and duodenum. Dr. Markham Skerritt commented upon the sub-acute course of cases of gallstone intestinal obstruction.—Mr. Lace alluded to two cases of gallstone obstruction of the intestine. In one of these there had been hæmatemesis, and the stone had been felt *per rectum*.

Dr. Theodore Fisher and Dr. Fortescue-Brickdale showed a specimen of **congenital hypertrophic stenosis** of the pylorus from a child aged 11 weeks. The stomach was not much dilated (capacity about 4 ounces), but the walls were thickened considerably. The œsophagus was dilated and hypertrophied. The pylorus showed the usual appearance of a hard, thick ring of muscular tissue, about three-quarters of an inch long, within which the mucous membrane was thrown into longitudinal folds. *Post-mortem*, water could not be forced through the pylorus. The intestines contained a little fæcal matter. The other organs were healthy. The child weighed 6 lb. 6 oz., and had vomited since it was a fortnight old. The vomiting was nearly constant in small amounts after every feed, but occasionally a much larger quantity was returned. The obstruction cannot have been complete during life as there was no constipation. The pyloric tumour could not be felt, but peristalsis was very apparent in the epigastric region. This, with the history and the emaciation, determined the diagnosis. The child died suddenly on the second night after admission to hospital.

Dr. E. W. Hey Groves read a paper on **the advantages of enucleation of the tonsils over their removal by the guillotine.** [This paper will shortly be published.] Dr. Watson Williams said that he could not assent to the statement that the tonsils were functionally useless structures. He believed that the ring of lymphoid tissue consisting of the faucial, lingual, and naso-pharyngeal tonsils, so well developed in the earlier years of life, were probably of greater functional ability than often was supposed, and that while these lymphoid tissue aggregations might be of some as yet unknown useful purpose in life, there was strong evidence that when healthy they were protective. Invading micro-organisms were arrested there, and infective processes leading to tonsillitis and so forth were in many instances thus limited in their action instead of resulting in systemic invasion. It was at one time presumed that as we were ignorant of any important function appertaining to such—that the spleen was of no use; and even comparatively recently the thyroid gland was presumed to be of no service to the organism. Hence sweeping generalisation and assertions that the tonsils were useless structures were to be accepted with great reserve. Another point that he felt was of great importance was the influence of chronic enlargement of the tonsils in the so-called quiescent phases, for though it was readily appreciated that acute tonsillitis was painful and a grave source of ill-health at times, he was of opinion that during the intervals between these acute exacerbations these tonsils maintained a condition of persistent though perhaps slight sapræmia, with consequent anæmia and inhibited tissue metabolism, the patient being listless and lacking in vitality. The remarkable change for the better resulting from removal of the tonsils when they were diseased were a fairly convincing proof of this. As regards the value of enucleation as compared with tonsillotomy, he felt that if the tonsils were useful structures, Dr. Groves's main contention in favour of the more radical procedure fell to the ground. He also maintained that if the Matthieu tonsil guillotine was used the results were more consistently satisfactory than with the use of the Mackenzie tonsillotome and its various modifications. —Mr. H. F. Mole said that he had to operate on a large number of cases of enlarged tonsils. He always used the guillotine, and the results were as a rule very satisfactory. He thought the operation of enucleation should be reserved for two classes of cases: first, those in which, after removal of the tonsils by the guillotine, the patients were liable to recurring attacks of tonsillitis; secondly, those patients with flat or buried tonsils which did not project beyond the pillars of the fauces and could not be engaged in the ring of a guillotine, and who were also liable to recurring attacks of tonsillitis. Both these classes of cases were usually found in adults, the second entirely so, and these latter could almost as satisfactorily be dealt with by the galvano-cautery or punch forceps in two or three sittings under cocaine. The great risk of the operation on tonsils lay in the anæsthetic, and that operation which, giving reasonably satisfactory results, could be performed under the shortest and least deep anæsthesia was obviously the best. The operation of enucleation required a longer and deeper anæsthesia than the guillotine operation, and its universal adoption, in addition to being unnecessary, would very greatly increase the mortality of the operation. He thought it should be limited to the cases mentioned. He thought it unwise to state that the tonsil was a useless structure. It was possible that it might perform a very important purpose in arresting the descent of micro-organisms to the respiratory or alimentary tract.

J. LACY FIRTH and
HAROLD F. MOLE (*Hon. Sec.*).

### Local Medical Notes.

